# Knowledge, Attitude, and Practice (KAP) Status toward Clinical Reasoning and Evidence-Based Medicine among the Medical Interns and Gynecology Residents of Iran University of Medical Sciences

**DOI:** 10.1155/2024/6546432

**Published:** 2024-03-13

**Authors:** Mojgan Javedani Masroor, Saeid Rezaei, Seyed Ali Hashemi Kiapey, Mahdi Hakiminezhad, Seyyed Amir Yasin Ahmadi

**Affiliations:** ^1^Center for Educational Research in Medical Sciences (CERMS), Department of Medical Education, School of Medicine, Iran University of Medical Sciences, Tehran, Iran; ^2^Shahid AkbarAbadi Clinical Research Development Unit (SHACRDU), School of Medicine, Department of Obstetrics and Gynecology, Iran University of Medical Sciences, Tehran, Iran; ^3^Eye and Skull Base Research Centers, The Five Senses Institute, Rassoul Akram Hospital, Iran University of Medical Sciences, Tehran, Iran; ^4^Department of Surgery, Imam Reza Hospital, Mashhad University of Medical Sciences, Mashhad, Iran; ^5^Firoozgar Clinical Research Development Center (FCRDC), Department of Surgery, Iran University of Medical Sciences, Tehran, Iran; ^6^Preventive Medicine and Public Health Research Center, Psychosocial Health Research Institute, Iran University of Medical Sciences, Tehran, Iran

## Abstract

**Background:**

Clinical reasoning and evidence-based medicine (EBM) are important concepts in modern medicine.

**Objective:**

We performed this study to investigate the knowledge, attitude, and practice (KAP) status toward clinical reasoning and EBM among the medical interns and gynecology resident physicians of Iran University of Medical Sciences and related factors.

**Methods:**

A cross-sectional study (Tehran, Iran, first half of 2022) was conducted based on two researcher-made questionnaires consisting of three components for each including clinical reasoning attitude (CR-A), clinical reasoning knowledge (CR-K), clinical reasoning practice (CR-P), EBM attitude (EBM-A), EBM knowledge (EBM-K), and EBM practice (EBM-P). The related factors were age, gender, educational level, score of general practice education, having research experience, and general practice experience.

**Results:**

A total of 60 individuals participated. The mean score was good for CR-A, moderate for CR-K, moderate for CR-P, good for EBM-A, moderate for EBM-K, and moderate for EBM-P. The total score was moderate in both clinical reasoning and EBM. Among the related factors, CR-P was associated with higher educational levels and having experience in general practice (*P*  < 0.05). Research experience was associated with better CR-K and all KAP components for EBM (*P*  < 0.05).

**Conclusion:**

The total score and many of the KAP components had moderate status for clinical reasoning and EBM. Planning on the associated factors should be regarded in the future. Such questionnaires are suggested to be validated for use in quasi-experimental studies.

## 1. Introduction

Clinical reasoning is an approach used to solve clinical problems to achieve a correct diagnosis [1]. In this approach, that begins at the same time as the medical history is taken, the physician or medical student uses statistical attitude and critical thinking to summarize the problems (problem list), hypothesize using differential diagnosis, and test their hypotheses by re-examination, history, additional questions and examinations, and finally requesting appropriate paraclinical tests. This approach is accompanied by a comprehensive view of the patient, such as anatomical localization of the patient's problems and also attention to life-threatening diagnoses, and helps observe professional ethics [2]. Obviously, such a process requires mathematics, statistics, and quantification methods. Therefore, over time, due to the need to integrate statistics with clinical medicine, an approach called clinical epidemiology was formed, which has been more commonly known as evidence-based medicine (EBM) since 1981 [3].

These approaches can now be used to solve patient-related issues, which are called clinical reasoning. One of the theories on which clinical reasoning and also EBM are based is Bayes statistical theory. In Bayes statistical theory, the probability of a phenomenon is propounded instead of the approach of being/not being in that phenomenon and this probability is also expressed as prior and posterior, which are now referred to as pretest probability and posttest probability [4].

Experts believe that the use of the Bayesian approach in medicine has led to a reduction in unnecessary tests and an increase in necessary tests because the goal is to reach from the probability of pretest to the probability of posttest by minimal material and spiritual costs [5]. In clinical reasoning, there is a possibility of pretesting for each hypothesis (differential diagnosis) which is estimated using semiquantitative methods (such as the rule of thumb). Two thresholds of test (test threshold) and treatment (treatment threshold) are defined for this probability range. If the estimated probability reaches the test threshold, the hypothesis test is performed and if it reaches the treatment threshold, treatment is performed [2]. This process is repeated in cycles, and each posttest probability becomes a pretest probability for the next step. Although this cycle apparently stops when the diagnosis and treatment thresholds are reached, the process of clinical reasoning continues so that other hypotheses, concurrent diagnoses, follow-up of treatment complications in previous visits, etc., are not lost.

Despite the importance of clinical reasoning in medicine and clinical practice, there is no information about the knowledge, attitude, and practice (KAP) of the physicians and medical students toward it. We performed this study to investigate the KAP status toward clinical reasoning and EBM among the medical interns and gynecology resident physicians of Iran University of Medical Sciences and the related factors and predictors.

## 2. Materials and Methods

### 2.1. Study Design

A cross-sectional study was conducted based on researcher-made questionnaires. The questionnaires were validated to be used in this cross-sectional report. The study protocol was approved by the ethics committee of Iran University of Medical Sciences with registration number IR.IUMS.REC.1400.1224.

### 2.2. Samples

The reference population included all the gynecology interns and residents in Iran. Medical interns and gynecology residents of Shahid Akbar-Abadi Hospital, Tehran, Iran, during the first half of 2022 were our accessible population. Eligibility criteria other than the criteria of reference population were lack of major educational problems, complete filling of the questionnaire, and giving informed consent. The samples were selected using Quota sampling from different educational levels. Sample size was calculated for Cronbach's alpha estimation (H0: 0.70, H1: 0.85, items = 10 for each component, type I error = 0.05, and power = 95%) as 61 individuals in PASS11 software (NCSS, LLC. Kaysville, Utah, USA).

### 2.3. Study Tools

Two researcher-made questionnaires were used consisting of 6 components (3 components for each) including clinical reasoning attitude (CR-A), clinical reasoning knowledge (CR-K), clinical reasoning practice (CR-P), EBM attitude (EBM-A), EBM knowledge (EBM-K), and EBM practice (EBM-P). To score each component, the mean of Likert points of its questions was reported (from −2 to +2). To score the whole status of clinical reasoning and EBM for each, the component scores were added to each other (from −6 to +6).

The preparation and validation process of this questionnaire was presented in the local language [6]. Briefly, after preparation of the questionnaires, it was validated by an expert panel (*n* = 10) using the content validity index (CVI) and content validity ratio (CVR) and then reliability analysis for internal consistency and confirmatory factor analysis were performed. The questions of the components were based on self-reporting and trusting the honesty of the participants. For attitude, the questions started with “in my opinion” and the answers were “strongly disagree” (−2), “disagree” (−1), “no opinion” (0), “agree” (+1), and “strongly agree” (+2). For knowledge, the questions started with “how much are you familiar/you agree with the following statement” and the answers were “about 0%” (−2), “about 25%” (−1), “about 50%” (0), “about 75%” (+1), and “about 100%” (+2). For practice, the questions started with “what percent of the occasions do you do following statement” and the answers were “about 0%” (−2), “about 25%” (−1), “about 50%” (0), “about 75%” (+1), and “about 100%” (+2). The English translation of these questionnaires is attached (supplementary Tables S1 and S2).

In the final validated version of the questionnaires, there were a total of 39 items (out of 60 items of the primary version) with 5 choices on the Likert scale. For each component, Cronbach's alpha was as follows: CR-A (0.840), CR-K (0.767), CR-P (0.765), EBM-A (0.844), EBM-K (0.918), and EBM-P (0.876). Total Cronbach's alpha was 0.875 for clinical reasoning and 0.932 for the EBM questionnaire. The reasons of item removal from the primary version were lack of validity as well as low internal consistency based on item-total correlation (stepwise removal to reach the highest Cronbach's alpha).

Other than the mentioned questionnaires, some baseline characteristics were evaluated including age, gender, educational level, mean score of general practice education, having research experience, and general practice experience.

### 2.4. Variables and Definitions

KAP components: all the parts of the questionnaires including CR-A, CR-K, CR-P, EBM-A, EBM-K, and EBM-P. Among them, CR-P was our most important practical goal in this study.

Component score: the mean of Likert points for each component (from −2 to +2). The qualitative classification is as −2 to −1.2 (very weak), −1.2 to −0.4 (weak), −0.4 to +0.4 (moderate), +0.4 to +1.2 (good), and +1.2 to +2 (very good).

Total score: addition of component scores for each of clinical reasoning and EBM to report the whole status of them (from −6 to +6). The qualitative classification is as −6 to −3.6 (very weak), −3.6 to −1.2 (weak), −1.2 to +1.2 (moderate), +1.2 to +3.6 (good), and +3.6 to +6 (very good).

Related factors: the baseline characteristics mentioned were considered as the factors related to clinical reasoning and EBM.

Educational level: consisted of five ordinal groups including intern, resident year 1, resident year 2, resident year 3, and resident year 4.

GP score: average score (mark) of the passed credits during being a student of doctor of medicine (general practice). This score was based on the Iranian system from 0 to 20 and we reported as ordinal groups including <14, 14 to 15, 15 to 16, 16 to 17, and more than 17.

Research experience: an ordinal variable with the groups including “I don't have [a research experience]”, “[I had merely] executive contribution,” “[I have] congress presentation [and I don't have journal publication]”, “[I had] journal article,” and “[I have] a lot of papers.”

General practice: having experience of practice in general medicine before starting residency. Obviously, interns were in a negative class of this variable.

### 2.5. Statistical Analysis

Descriptive statistics were used for baseline characteristics and questionnaire scores (component and total). To find the associations of component scores with the related factors, independent *t*-test and analysis of variance (ANOVA) were used. In the cases of ANOVA, if the *F* test *P* value was <0.2, Dunnett's post hoc test was performed. Pearson correlation analysis was used to investigate the bivariate correlation of the KAP components. In order to find the predictors of each KAP component based on the related factors, multiple linear regression was used. All the analyses were performed in SPSS 24 (IBM, US).

## 3. Results

### 3.1. Baseline Characteristics

A total of 60 individuals participated; about 78.3% were medical interns, and 21.7% were gynecology residents. The mean age was 26.43 ± 3.60 years, and about 43.3% of them were male. The GP score was more than 16 among 80% of them. About 46.7% did not have research experience. About 16.7% had experience in general practice. The complete baseline characteristics are shown in detail (Table 1).

### 3.2. Questionnaire Scores

**Figure 1:**
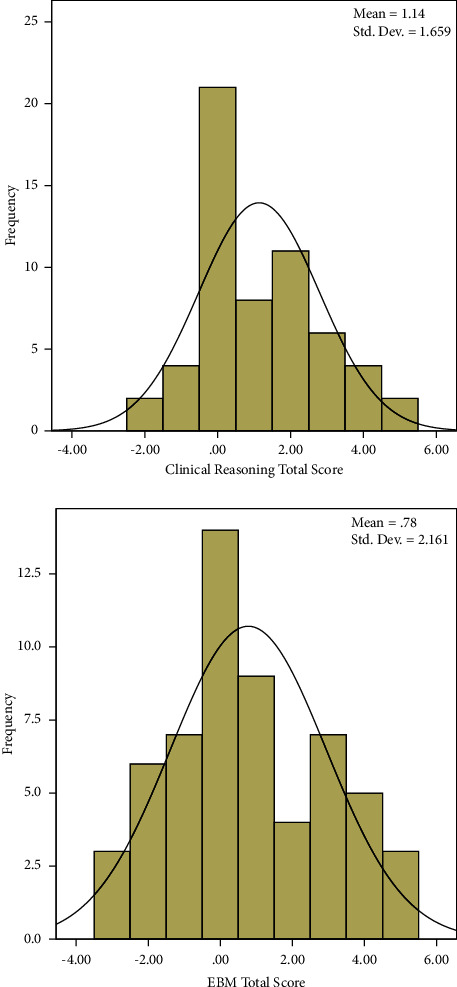
Distribution of clinical reasoning and EBM total score.

The detailed questionnaires and the factor loads are shown as supplementary materials (Tables S1 and S2). Among the investigated 3 components of clinical reasoning, the mean score was 1.12 for CR-A (good), −0.09 for CR-K (moderate), and 0.33 for CR-P (moderate). Among the investigated 3 components of EBM, the mean score was 0.85 for EBM-A (good), −0.26 for EBM-K (moderate), and 0.23 for EBM-P (moderate) (Table 2). The total score of clinical reasoning was 1.14 (moderate), and the total score of EBM was 0.78 (moderate) (Figure 1).

### 3.3. Effects of Related Factors

Associations of KAP component scores with baseline characteristics were studied. Accordingly, age and gender did not show significant associations with the scores (*P* > 0.05). For the educational levels, there was no significant variation in the scores between the levels in comparison to within group variation based on ANOVA (*P* > 0.05). Post hoc wise, year 3 residents, had 1.125 more scores than interns in CR-P (*P* = 0.049, *F* test *P* < 0.02). For the GP score, there was a significant variation in the CR-K scores between the groups of GP score in comparison to within group variation based on ANOVA (*P* = 0.024). However, no significant post hoc association was found (*P* > 0.05). For research experience, there were significant variations in the CR-K, EBM-A, EBM-K, and EBM-P scores for each between the groups of research experience in comparison to within group variation based on ANOVA (*P* = 0.025 for EBM-A, *P* < 0.001 for others). Post hoc wise, there were some significant post hoc associations in favor of higher levels of research experience (*P* < 0.05). For experience of general practice, the participants having this experience had 1.034 more scores in CR-P (*P* < 0.001). In addition to the mentioned associations, some other associations were significant at 0.05< *P* < 0.1 (Table 3).

### 3.4. Correlation of KAP Components

Bivariate correlation of the 6 KAP components was studied, and most comparisons showed positive and significant correlation (*P* < 0.05). The greatest correlation was between EBM-K and EBM-P (*r* = 0.701, *P* < 0.001) (Table 4).

### 3.5. Predictors of KAP Components

According to the multiple linear regression model based on baseline characteristics, all the KAP components (except CR-A) were predictable. Considering CR-P as the most practical component among them, it could be predicted by the GP score (positive coefficient), research experience (positive coefficient), and general practice experience (positive coefficient). The details are shown in Table 5.

## 4. Discussion

### 4.1. Summary of Results

The present study was performed to investigate both KAP status and the related factors in clinical reasoning and EBM. Briefly, the descriptive results for age, gender, and educational level were approximately as what expected. Lack of volunteer participation of any resident in year 4 was notable. Eighty percent of the participants had high GP scores. It seemed that our top students might have a greater tendency for volunteer participation in this study. Hence, it might cause bias for our conclusions. As expected, about half of the participants did not have research experience. Thirty percent of the participants had at least one journal paper. According to our field investigations (not documented), medical students with experience of publishing journal papers seemed to be less than this percentage. Again, it seemed that researchers might have a more tendency for volunteer participation in this study.

For the component scores, attitude-related scores had higher points followed by practice- and knowledge-related scores. This might result from more difficult questions of knowledge or due to a real low level of knowledge among the participants. Only attitude-related components had good status, while other components and total scores had moderate status. Higher attitude levels of the participants could indicate their interest in improving their low knowledge. Among the KAP components, CR-P was the most important practical goal in our study as we ideologically wanted to reach to a point in which the physicians would have an acceptable quality of practice in clinical reasoning.

From the inferential point of view, CR-A did not show association with the related factors as it was not predictable in regression modeling. CR-K was associated with experience of paper publication in journals. It seemed that the similar topics between clinical reasoning and research principles resulted in this finding. CR-P was associated with a higher educational level and having experience of general practice. The reason could be this fact that what we were looking for to improve CR-P, a part of it became during years of clinical experience. All EBM components were associated with research experience. It is obvious that there are a lot of similarities between EBM and research.

Pairwise comparison of KAP components was performed. The significant correlations with CR-P, as the most important practical goal of the study, were in the range of 0.316 to 0.571, and all of them were statistically significant (*P* < 0.05, H0: *r* = 0). Considering CR-P as the outcome, these significant correlations could indicate a content validity in our questionnaire. In addition, it indicated that improvement in the KAP components among students might result in the improvement in CR-P; the most effective variables were EBM-P (*P* < 0.001, *r* = 0.571) and CR-A (*P* < 0.001, *r* = 0.528). From the view point of discriminant validity, the best condition for such a correlation matrix was the existence of significant correlations neither very low nor very high [7, 8].

According to the results of multiple regression modeling, CR-A was not predictable by these related factors. However, CR-K, CR-P, EBM-A, EBM-K, and EBM-P could be explained by the related factors (*R*^2^ range: 0.189–0.438). The most interesting finding was the negative role of age in EBM-P. This might be due to recent emphases on EBM in medical education in comparison to the graduates of previous generations.

### 4.2. Literature Review

To date, EBM and clinical reasoning have not been studied beside each other as a whole similar to the present study. However, some relatively similar studies are noted. Seemingly, most of the studies conducted about EBM. These studies have already been summarized in a systematic review by Ghojazadeh et al. as well as Barzkar et al. [9, 10]. The latter study has been registered in the Cochrane database.

Van Gessel et al. in Switzerland examined clinical reasoning in students after a 12-week course entitled “Introduction to Clinical Reasoning.” They used a researcher-made questionnaire with 10 questions. Finally, 124 participants completed the questionnaire and concluded that this training course can be effective for better transferring students from preclinical to clinical levels [11].

Park et al. in South Korea examined the relationship between the Objective Structured Clinical Examination (OSCE) score and clinical reasoning ability. Sixty-five 4^th^ year medical students were included in the study. To score clinical reasoning, students were asked to write a differential diagnosis for four patients, and then their sheets were scored by two separate physicians. They found that clinical reasoning was related to GPA scores and right patient diagnosis. However, they found no correlation between clinical reasoning and OSCE scores. They suggested that the evaluation of clinical reasoning in the OSCE exam should be strengthened so that its score would indicate clinical reasoning skills [12].

Moghadami et al. in Shiraz, Iran, in a clinical trial examined the effect of disease scripting method (disease script; a method that for each disease mentions its theoretical points such as etiology, pathophysiology, semiology, diagnosis, and treatment) on clinical reasoning skills in 100 4^th^ year medical students. They found that this method increased the score and increased clinical reasoning skills. Their study tool was the text concordance test, which was a tool for measuring clinical reasoning skills [13]. In this questionnaire, some clinical examples are given in such a way that if we think about a particular diagnosis and our clinical or paraclinical finding is something, with what score (Likert scale from 2− to +2) our diagnosis is probable. In our study, instead of thematic skills with clinical examples, attention has been paid to knowledge, attitude, and practice toward the principles of clinical reasoning. Therefore, our results may be generalized to many thematic issues and examples.

Rashidbeygi and Sayehmiri in Ilam, Iran, examined the knowledge and attitude of physicians toward EBM. A researcher-made questionnaire was used for 94 physicians. They found that younger physicians, as well as specialist and subspecialist physicians, were more familiar than older physicians and general practitioners [14]. This might have resulted from the propagation of teaching EBM in universities during recent years.

Sadeghi et al. in Kerman, Iran, studied the knowledge, attitude, and application of EBM in 94 residents. Although more than 80% of respondents found EBM useful and were interested in it, only about 5% of them stated that they use it in their clinical work [15]. Sahebalzamani et al. in Tehran, Iran, examined the knowledge, attitude, and ability toward EBM in 40 residents. Their awareness was moderate, and their ability was assessed as unfavorable [16]. Salehifar et al. in Alborz, Iran, studied the knowledge, performance, and attitude of 40 clinical faculty members. About 90% of them believed that EBM would play an effective role in improving the quality of patient care. However, many of them stated that their knowledge in this field was low or medium [17]. Ghojazadeh et al. in a systematic review examined the knowledge and attitude toward EBM and its obstacles in Iran. Familiarity with the term EBM was less than 50%, and the most used resource was a reference book (text book). Obstacles were lack of English language proficiency, physician noncooperation, lack of authority to make changes, lack of skills in research methods, and insufficient time to study [10].

Beside clinical reasoning and EBM, other concepts have also been studied in medical education. Mamede et al. in the Netherlands studied the effect of using the structured reflection method on the diagnostic accuracy score in 110 4^th^ year medical students and concluded that this method, in addition to the effect it had on theoretical training, caused better clinical performance [18]. In Canada, Chamberland et al. examined the effect of the self-explanation method on diagnostic performance in 54 third-year medical students. They concluded that self-explanation leads to better learning of clinical reasoning and enhances diagnostic performance [19]. Chamberland et al. had emphasized the use of this method in various schemes [20, 21] as Mamede et al. had emphasized reflective methods [22, 23].

### 4.3. Strengths and Limitations

It was the first time that clinical reasoning and EBM questionnaires were investigated in one study. This approach had some threats and opportunities mentioned above. The most important one was to discriminate the KAP components along with a simultaneous association between each other. We had designed the questions based on the self-reporting method as this method had been used before [24]. This method has some pros and cons. Using self-reporting statements instead of thematic examples might result in the spread of its use beyond the region of study. Nevertheless, trusting the participants might not be as good as examining them. A small sample size was another limitation. However, many studies mentioned above had similar sample sizes. Although validity and reliability were investigated, the purpose of this study was not psychometric tool development, but it was a cross-sectional report. The questionnaires should be redesigned and reevaluated for each specific study.

## 5. Conclusions

The present study investigated KAP status in clinical reasoning and EBM along with each other among medical interns and gynecology residents. Accordingly, the attitudes toward clinical reasoning and EBM were good, while knowledge and practice toward them were moderate. The overall status in clinical reasoning and EBM was moderate. Among the related factors, CR-P was associated with higher educational levels and having experience in general practice. Research experience was associated with better CR-K and all KAP components for EBM. To improve CR-P status, as the practical goal of this study, in physicians, planning for improvement in the other KAP components especially EBM-P is suggested. These questionnaires are suggested to be validated for use in quasi-experimental studies after psychometric studies in other populations.

## Figures and Tables

**Table 1:** Descriptive results of the related factors among the participants.

Variable (unit)	Frequency/mean (SD)
Age (years)	26.43 (3.60)
Gender (male)	43.3%
Educational level	
Intern	78.3%
Resident (year 1)	6.7%
Resident (year 2)	8.3%
Resident (year 3)	6.7%
Resident (year 4)	0.0%
GP score	
<14	5%
14-15	5%
15-16	15%
16-17	36.7%
>17	38.3%
Research experience	
I don't have	46.7%
Executive contribution	18.3%
Congress presentation	5%
Journal article	25%
A lot of papers	5%
General practice (I had)	16.7%

**Table 2:** Descriptive results of the KAP components.

KAP component	Mean (median)	SD (range)
CR-A	1.12 (1.00)	0.56 (2.57)
CR-K	−0.09 (−0.20)	1.02 (4.00)
CR-P	0.33 (0.36)	0.90 (3.86)
EBM-A	0.85 (0.86)	0.63 (2.57)
EBM-K	−0.26 (−0.38)	1.19 (4.00)
EBM-P	0.23 (0.20)	1.08 (3.80)

SD: standard deviation.

**Table 3:** Association of the related factors with the KAP components.

Variable (unit)	Statistical test	KAP component (score from −2 to 2)					
		CR-A	CR-K	CR-P	EBM-A	EBM-K	EBM-P
Age (years)	Pearson corr.						
	Coef. (*r*)	−0.025	0.062	0.251	−0.120	−0.128	−0.081
	*P* value	0.847	0.637	0.057^#^	0.361	0.334	0.545

Gender (male)	Independent *t*^1^						
	*P* value	0.031	−0.132	−0.123	−0.069	−0.506	−0.388
	Mean diff.	0.832	0.625	0.611	0.676	0.108	0.179

Educational level	ANOVA^2^						
	F stat.	0.757	0.450	2.139	0.588	1.287	1.612
	*P* value	0.523	0.718	0.136	0.625	0.288	0.197

Intern	Mean diff.	NA^3^	NA	0.000	NA	NA	0.000
	*P* value			Ref.			Ref.

Resident (year 1)	Mean diff.	NA	NA	−0.053	NA	NA	−0.898
	*P* value			0.999			0.291

Resident (year 2)	Mean diff.	NA	NA	0.282	NA	NA	−0.498
	*P* value			0.869			0.684

Resident (year 3)	Mean diff.	NA	NA	1.125	NA	NA	0.522
	*P* value			0.049^*∗*^			0.682

GP score	ANOVA						
	F stat.	0.502	3.071	1.163	1.574	2.068	2.193
	*P* value	0.735	0.024^*∗*^	0.338	0.194	0.098^#^	0.082^#^

<14	Mean diff.	NA	0.000	NA	0.000	0.000	0.000
	*P* value		Ref.		Ref.	Ref.	Ref.

14-15	Mean diff.	NA	0.733	NA	0.571	1.375	0.333
	*P* value		0.644		0.511	0.319	0.964

15-16	Mean diff.	NA	0.488	NA	0.603	1.490	1.342
	*P* value		0.765		0.317	0.145	0.144

16-17	Mean diff.	NA	0.139	NA	0.482	0.850	0.333
	*P* value		0.994		0.424	0.469	0.915

>17	Mean diff.	NA	1.084	NA	0.812	1.572	0.945
	*P* value		0.163		0.091^#^	0.076^#^	0.306

Research experience	ANOVA						
	*F* stat.	1.879	5.100	1.944	3.019	9.477	8.281
	*P* value	0.127	0.001^*∗*^	0.117	0.025^*∗*^	<0.001^*∗*^	<0.001^*∗*^

I don't have	Mean diff.	0.000	0.000	0.000	0.000	0.000	0.000
	*P* value	Ref.	Ref.	Ref.	Ref.	Ref.	Ref.

Executive contribution	Mean diff.	0.449	0.147	0.686	0.313	0.676	0.618
	*P* value	0.090^#^	0.983	0.119	0.440	0.178	0.190

Congress presentation	Mean diff.	−0.265	0.426	0.534	−0.098	1.521	0.496
	*P* value	0.880	0.892	0.768	0.998	0.041^*∗*^	0.813

Journal article	Mean diff.	0.220	1.106	0.620	0.625	0.512	1.470
	*P* value	0.593	0.001^*∗*^	0.116	0.007^*∗*^	<0.001^*∗*^	<0.001^*∗*^

A lot of papers	Mean diff.	0.258	1.560	0.653	0.282	2.750	2.063
	*P* value	0.890	0.025^*∗*^	0.758	0.888	0.001^*∗*^	0.009^*∗*^

General practice (I had)	Independent t						
	Mean diff.	0.134	0.084	1.034	−0.098	−0.489	0.325
	*P* value	0.494	0.815	<0.001^*∗*^^4^	0.689	0.241	0.390

(1) Positive mean difference indicates higher mean in males and those having experience of general practice. (2) Dunnett post hoc test was performed in which positive mean difference indicates higher mean in comparison to the reference category. (3) Not applicable due to *F* test *P* value >0.2. (4) Unequal variance was assumed. ^*∗*^Significant at 0.05. ^#^Significant at 0.1.

**Table 4:** Bivariate correlation of the KAP components.

KAP component	CR-A	CR-K	CR-P	EBM-A	EBM-K	EBM-P
CR-A	1					

CR-K	0.202^1^	1				
	0.122^2^					

CR-P	0.528	0.438	1			
	<0.001^*∗*^	0.001^*∗*^				

EBM-A	0.472	0.349	0.316	1		
	<0.001^*∗*^	0.006^*∗*^	0.016^*∗*^			

EBM-K	0.129	0.377	0.382	0.533	1	
	0.331	<0.001^*∗*^	0.003^*∗*^	<0.001^*∗*^		

EBM-P	0.352	0.571	0.571	0.492	0.701	1
	0.007^*∗*^	<0.001^*∗*^	<0.001^*∗*^	<0.001^*∗*^	<0.001^*∗*^	

(1) Pearson correlation coefficient and (2) *P* value. ^*∗*^Significant at *P* < 0.05.

**Table 5:** Linear regression analysis was used to predict the score of the KAP components based on the related factors.

Variable (unit)	Regression results	CR-A	CR-K	CR-P	EBM-A	EBM-K	EBM-P
Model	Constant	Not predictable	−1.305	−0.778	0.192	−1.596	2.128
	*R* ^2^		0.331	0.343	0.189	0.438	0.429
	Adjusted *R*^2^		0.308	0.307	0.161	0.418	0.397

Age (year)	*Beta* coef. (95% CI)						−0.100 (−0.193–−0.006)
	*P* value						0.037

GP score (ordinal, *per* increasing one group)	*Beta* coef. (95% CI)		0.250 (0.048–0.453)	0.233 (0.048–0.418)	0.153 (0.016–0.290)	0.229 (0.011–0.446)	
	*P* value		0.016	0.014	0.030	0.040	

Research experience (ordinal, *per* increasing one group)	*Beta* coef. (95% CI)		0.380 (0.220–0.539)	0.177 (0.031–0.324)	0.160 (0.051–0.268)	0.551 (0.375–0.727)	0.468 (0.304–0.631)
	*P* value		<0.001	0.019	0.005	<0.001	<0.001

General practice (I had)	*Beta* coef. (95% CI)			1.150 (0.616–1.684)			1.003 (0.104–1.903)
	*P* value			<0.001			0.029

All the remaining covariates are significant at 0.1 according to the backward method. Gender and educational level could not predict any of the outcomes. CI: confidence interval.

## Data Availability

Data are available by the corresponding author upon reasonable request.

## Supplementary Materials

Supplementary Materials Table Supplementary 1 shows the components and items of the clinical reasoning questionnaire and related statistical information. Table Supplementary 2 shows the components and items of the EBM questionnaire and related statistical information.
