# Supplementary material for: Knowledge, Attitude, and Practice (KAP) Status toward Clinical Reasoning and Evidence-Based Medicine among the Medical Interns and Gynecology Residents of Iran University of Medical Sciences
Source: ScientificWorldJournal. 2024 Mar 13;2024:6546432. doi: 10.1155/2024/6546432 (PMC10954360; doi:10.1155/2024/6546432)
Supplement: Supplementary Materials — Table Supplementary 1 shows the components and items of the clinical reasoning questionnaire and related statistical information. Table Supplementary 2 shows the components and items of the EBM questionnaire and related statistical information. [file 6546432.f1.zip › S1 (2).docx]

Table S1) Components and items of clinical reasoning questionnaire, and related statistical information.

| **Component (primer context)** | **Question (item) context** | **Factor load^5^** | **Cronbach alpha** |
| --- | --- | --- | --- |
| **Attitude**  (in my opinion, clinical reasoning …)^1^ | Impedes misdirection during approach to a patient | 0.653 | 0.840 |
|  | Helps considering the other simultaneous diagnoses^4^ | 0.888 |  |
|  | Causes identification of life-threatening diseases | 0.862 |  |
|  | Causes reduction of unnecessary paraclinical evaluations | 0.605 |  |
|  | Causes improvement in systematic and critical thinking | 0.506 |  |
|  | Is like a bridge between EBM and clinical actions | 0.552 |  |
|  | Should be in educational curriculum | 0.721 |  |
| **Knowledge**  (how much were you familiar or how much do you agree)^2^ | I am familiar with the concepts of pretest and posttest probabilities | 0.553 | 0.767 |
|  | I am familiar with test threshold and treatment threshold | 0.617 |  |
|  | I knew that creation and testing a hypothesis procedure is repeated as a cycle | 0.788 |  |
|  | I knew that pretest probability influenced predictive values | 0.641 |  |
|  | I knew that extraction of statistical information from articles might be needed | 0.567 |  |
| **Practice**  (How often [percentage] do you act)^3^ | I write differential diagnosis (DDx) in patients' medical documents | 0.570 | 0.865 |
|  | I localize the problems anatomically if possible and make a DDx based on it | 0.619 |  |
|  | I act to summarize the main problems of a patient | 0.713 |  |
|  | I act continuously during evaluation of a patient to create hypothesis based on problems | 0.688 |  |
|  | I select the most appropriate diagnostic test for my hypothesis | 0.660 |  |
|  | I give importance to medical ethics and share my plans with a patient | 0.768 |  |
|  | I use clinical reasoning also for patient cares, future visits and follow-ups | 0.795 |  |

1) The choices were from strongly disagree or strongly agree. 2) The choices were 0%, 25%, 50%, 75% and 100% where 0% meant "I have never heard this concept or statement" and 100% meant "I have learnt in details and can teach to others". 3) The choices were 0%, 25%, 50%, 75% and 100%. 4) Some patients may have more than one definite diagnosis at the same time; for example, a patient with abdominal pain may have simultaneously both cholelithiasis and appendicitis. The sentence of this item is completely comprehensible in Persian language. 5) Based on confirmatory factor analysis using maximum likelihood method in Stata 14 (Stata Corp. LLC, USA). The first item of each component was the marker. Total R square was 0.996. Kaiser-Meyer-Olkin (KMO) = 0.755. Bartlett’s test of sphericity: P <0.001. Comparative fit index (CFI) = 0.783. Tucker-Lewis Index (TLI) = 0.751.
