# Supplementary material for: Knowledge, Attitude, and Practice (KAP) Status toward Clinical Reasoning and Evidence-Based Medicine among the Medical Interns and Gynecology Residents of Iran University of Medical Sciences
Source: ScientificWorldJournal. 2024 Mar 13;2024:6546432. doi: 10.1155/2024/6546432 (PMC10954360; doi:10.1155/2024/6546432)
Supplement: Supplementary Materials — Table Supplementary 1 shows the components and items of the clinical reasoning questionnaire and related statistical information. Table Supplementary 2 shows the components and items of the EBM questionnaire and related statistical information. [file 6546432.f1.zip › S2 (2).docx]

Table S2) Components and items of EBM questionnaire, and related statistical information.

| **Component (primer context)** | **Question (item) context** | **Factor load^5^** | **Cronbach alpha** |
| --- | --- | --- | --- |
| **Attitude**  (in my opinion, …)^1^ | Using EBM improves management of patients | 0.608 | 0.844 |
|  | Using EBM results in reduction of health system costs | 0.849 |  |
|  | Learning EBM is necessary for physicians | 0.956 |  |
|  | EBM should be in educational curriculum | 0.899 |  |
|  | The course of medical statistics is necessary for medical students | 0.555 |  |
|  | Familiarity with methodology and searching skill is necessary for physicians | 0.445 |  |
|  | A good doctor should not be necessarily familiar with principles of research^4^ | 0.305 |  |
| **Knowledge**  (how much were you familiar or how much do you agree)^2^ | I am familiar with concepts like relative risk, odds ratio and confidence interval | 0.656 | 0.918 |
|  | I knew that what were systematic review and meta-analysis | 0.756 |  |
|  | I can covert a patient's problem to a formulated question (PICO) | 0.749 |  |
|  | I knew levels of evidence | 0.840 |  |
|  | I knew domains of EBM (types of questions) | 0.851 |  |
|  | I am familiar with interpretation of statistical results in a paper | 0.777 |  |
|  | I knew the critical appraisal checklists of different studies | 0.758 |  |
|  | I am familiar with sources like PubMed, Cochrane and Up to Date | 0.700 |  |
| **Practice**  (How often [percentage] do you act)^3^ | I use the sources like PubMed, Cochrane and Up to Date for my educational aims | 0.779 | 0.876 |
|  | I use the sources like PubMed, Cochrane and Up to Date for my research aims | 0.724 |  |
|  | If necessary, I completely read a paper and interpret it | 0.857 |  |
|  | I keep my information updated | 0.761 |  |
|  | I participate in congresses, journal clubs etc. voluntarily out of my interest | 0.723 |  |

1) The choices were from strongly disagree or strongly agree. 2) The choices were 0%, 25%, 50%, 75% and 100% where 0% meant "I have never heard this concept or statement" and 100% meant "I have learnt in details and can teach to others". 3) The choices were 0%, 25%, 50%, 75% and 100%. 4) This item had reverse scaling (during analysis, point -2 was replaced with +2 and likewise the other points). 5) Based on confirmatory factor analysis using maximum likelihood method in Stata 14 (Stata Corp. LLC, USA). The first item of each component was the marker. Total R square was 0.999. Kaiser-Meyer-Olkin (KMO) = 0.845. Bartlett’s test of sphericity: P <0.001. Comparative fit index (CFI) = 0.835. Tucker-Lewis Index (TLI) = 0.812.
